# Mechanical lower back pain and sacroiliac joint dysfunction in golfers at two golf clubs in Durban, South Africa

**DOI:** 10.4102/sajp.v74i1.402

**Published:** 2018-03-28

**Authors:** Siyabonga H. Kunene, Hlengiwe Luthuli, Mthandeni Nkosi, Maqsood Haffejee, Iftikaar Jooma, Scott Munro

**Affiliations:** 1 Department of Physiotherapy, University of the Witwatersrand, South Africa; 2 Department of Physiotherapy, University of KwaZulu-Natal, South Africa

## Abstract

**Background:**

Mechanical lower back pain (MLBP) and sacroiliac joint dysfunction (SIJD) are common problems among golfers. There are currently few studies on golfers regarding the relationship between MLBP and SIJD.

**Objective:**

The objective of this study was to determine the prevalence of MLBP and SIJD and their association in golfers at two golf clubs in Durban, South Africa.

**Method:**

A correlation design included convenience sampling. The Standardised Nordic Questionnaire for the analysis of musculoskeletal symptoms determined the prevalence of MLBP. Sacroiliac joint pain provocative tests determined the prevalence of SIJD. Institutional ethical clearance was granted and consent from participants was obtained. Data were collected over 3 weeks and SPSS was used to calculate descriptive and inferential statistics.

**Results:**

There were 271 participants dominated by males (86.7%) aged between 39 and 47 years (33.2%). A total of 123 (45%) of the participants presented with MLBP and 62 (23%) with SIJD. The MLBP prevalence was moderately associated with age (*χ*^2^ = 71.22, *p* = 0.004) and years of experience (*χ*^2^ = 69.93, *p* = 0.001). The SIJD prevalence was moderately associated with age (*χ*^2^ = 55.49, *p* = 0.003) and poorly associated with years of experience (*χ*^2^ = 44.93, *p* = 0.005). Twenty-two per cent (60) had both MLBP and SIJD and 54% (146) had neither. A strong association (*χ*^2^ = 88.234, *p* = 0.000) between MLBP and SIJD was observed.

**Conclusion:**

There was a high prevalence of MLBP and SIJD and a strong association between them. A comprehensive management approach is recommended for golfers with MLBP and SIJD.

**Clinical Implications:**

This study will provide valuable knowledge that will assist clinicians, especially physiotherapists, in their clinical management of golfers with MLBP and SIJD. Intervention studies are needed to address lower back and sacroiliac joint problems reported in this study.

## Introduction

Golf is a common sport enjoyed by many people around the world – some play it for recreational purposes, while others see it as a sports career option. Mechanical lower back pain (MLBP) is the most common injury among golfers accounting for up to 34.5% of all injuries globally, especially among amateurs (Finn 2013; McHardy, Pollard & Luo 2007; Parziale & Mallon 2006; Wadsworth 2007). Lower back pain conditions account for approximately 25% of all golf injuries (Batt 1992; Finn 2013; Gosheger et al. 2003; Lindsay & Vandervoort 2014), with an incidence rate between 18.2% and 54% (Finn 2013; Lindsay & Vandervoort 2014; McHardy et al. 2007).

The incidence of MLBP in male golfers is 25%–30% and 22%–25% in female golfers in South Africa according to Reed and Wadsworth (2010). Golfers with pre-existing MLBP are more likely to present with disabling golf-related pain (McHardy & Pollard 2005). Common lower back pain may include muscle strains, spondylolysis, vertebral and rib stress fractures, osteoporosis, facet irritation and disc herniations (Finn 2013).

Among golfers, the lower back region is the most vulnerable to injuries because it undergoes strenuous movement and muscular activity, especially while swinging the golf club (Bulbulian 2001; Finn 2013; McHardy & Pollard 2005; Lindsay & Vandervoort 2014). Lower back problems can occur owing to the powerful rotation and extension motion in the golf swing (Lindsay & Vandervoort 2014; McHardy & Pollard 2005). Poor body posture when hitting the golf ball contributes to back pain as there is more spine forward flexion and greater side flexion on the back swing (Finn 2013; Gluck et al. 2008; Lindsay & Vandervoort 2014). Golfers also present with reduced trunk flexion which in turn leads to rotation of the spine when swinging (Finn 2013; McHardy & Pollard 2005). Golfers who carry their own bags have twice the incidence of back, shoulder and ankle injuries compared with those who do not carry their bags (Hosea & Gatt 1996).

Sacroiliac joint dysfunction (SIJD) is one of the major contributing factors to MLBP accounting for approximately 16%–30% of cases (Cohen, Chen & Neufeld 2014; Kirkaldy-Willis & Bernard 1999; Vanelderen et al. 2010). The SIJD generally refers to aberrant position or movement of sacroiliac joint (SIJ) structures that may or may not result in pain (Laslett, et al. 2003, 2005). Sacroiliac joints act as shock absorbers when physically active and they provide stable support to the upper body (Vleeming et al. 2012). When the SIJ is dysfunctional, it affects the golfer’s biomechanics during golf activity, which may lead to lower back problems, localised tenderness over the joint itself, pain in the buttock or posterior thigh and asymmetry of pelvic landmarks (Reed & Wadsworth 2010).

There is continuous research being carried out regarding SIJD as a source of lower back pain (Kirkaldy-Willis & Bernard 1999; Laslett et al., 2003, 2005; Sembrano & Polly 2009; Weksler et al. 2007). Some researchers regard SIJD as the major contributing factor to lower back pain while some think it is not important or relevant (Laslett 2008; Maigne, Aivaliklis & Pfefer 1996; Waddell 1998; Weksler et al. 2007). There are only limited studies on the association between MLBP and SIJD among golfers. The purpose of this study was therefore to determine the prevalence of MLBP and SIJD and their association in golfers at two golf clubs in Durban, South Africa.

## Materials and methods

A cross-sectional correlation study was undertaken to determine the association between MLBP and SIJD. A sample of convenience from registered amateur golfers at two golf clubs in Durban, KwaZulu-Natal, participated in this study. A Raosoft statistical tool was used to calculate a sample size of 271 participants out of the 910 population taking into consideration a 95% confidence level, 5% margin of error and 50% response distribution. Participants included male and female golfers aged between 21 and 55 years with no history of trauma-related back and SIJ pain.

The Standardised Nordic Questionnaire for the analysis of musculoskeletal symptoms was used to subjectively determine the prevalence of mechanical back pain among participants. The reliability and validity of the questionnaire has been investigated, and it has a sensitivity of 96% (Kuorinka et al. 1987).

Demographic questions which included gender, age, race and golf experience were developed by the first author and included in the above questionnaire as Section A. Section B comprising the Standardised Nordic Questionnaires for the analysis of musculoskeletal symptoms included general questions which with the aid of a body diagram helped to identify the exact location of the lower back pain and any musculoskeletal pain experienced by participants.

Section B also asked more detailed questions about pain, including questions that sought to determine if the pain had any functional impact at home and at work and the duration of the pain. Before the data were collected, a pilot study was conducted among 27 golfers from the same population to ensure the content validity of the questionnaire. No corrections and modifications were indicated by the results of the pilot study.

To determine the SIJ function, four SIJ provocation tests (compression, destruction, thigh thrust and sacral thrust tests) were used to objectively screen for any dysfunction in the SIJ joints. The reliability and validity of these tests has been investigated with a sensitivity of 94% and specificity of 79% (Laslett et al. 2005). Participants who tested positive for at least three tests were considered as having SIJD accordingly (Laslett el at. 2005).

Potential participants were provided with information leaflets with details about the study and were invited to participate in the study. Participants, after agreeing to participate, were first given the questionnaire to complete and then were immediately screened for SIJD using the four provocation tests. A data collection sheet was used to record all the findings of each objective test done. The objective screening took approximately 10 min to complete for each participant. Two of the authors performed the tests independently once for each participant. The inter-rater reliability was found to be high (*α* > 0.8) using Kripperndorff’s alpha test (Hayes & Krippendorff 2007). Participants were allocated a unique code to ensure participant privacy and confidentiality. The collected data were secured to maintain confidentiality.

The data were captured using a Microsoft Excel spread sheet and later transferred to SPSS (version 22) software for analysis. Descriptive and inferential statistics were used to analyse the data. Descriptive statistics included the calculation of frequencies, ranges and cross-tabulations of variables. Inferential statistics included the calculation of Pearson Chi-Square and *p*-values for all categorical data. The alpha level was set at *p* < 0.05.

### Ethical consideration

Ethical clearance (BFC377/15) was obtained from the Biomedical Research Ethics Committee (BREC) of the University of KwaZulu-Natal. Permission to conduct the study was granted by the two golf club managers.

## Results

The study yielded a 100% response as all 271 golfers who participated in the study completed the questionnaire and physical screening. Males 235 (86.7%) made up the largest percentage compared to females 36 (13.3%) and 90 participants between ages 39 and 47 made up the highest percentage (33.2%) (Table 1). White people were the highest represented race at 131 (48.3%), and 116 (42.8%) of the participants had 11–20 years of golfing experience.

**TABLE 1:** Demographic profile (*n* = 271).

Demographics	Categories	*n*	%
Gender	Male Female	235 36	86.7 13.3
Age	21–29 30–38 39–47 48–55	29 75 90 77	10.7 27.7 33.2 28.4
Race	White African Indian Mixed race Asian	131 35 99 02 04	48.3 12.9 36.5 0.7 1.5
Golf experience	1–10 11–20 21–30 31–40	9 116 38 21	35.1 42.8 14.0 7.7

Table 2 outlines the prevalence of MLBP versus participants’ demographic profile. One hundred and twenty-three (45%) of the participants reported that they recently experienced MLBP. Nine (9%) reported that they were once hospitalised, 4 (3%) changed jobs or duties, 23 (19%) reduced their physical activity and 28 (23%) consulted a health professional owing to MLBP. There was a moderate relationship between the MLBP and age (*χ*^2^ = 71.22, *p* = 0.004) and golf experience (*χ*^2^ = 69.93, *p* = 0.001). There was also a poor relationship between participants who reported reduced activity owing to MLBP and golf experience (*χ*^2^ = 33.22 *p* = 0.004).

**TABLE 2:** Mechanical lower back pain versus participants’ demographic profile (*n* = 271).

Domains	Prevalence (%)	Gender	Age	Race	Experience
Currently experienced MLBP	123 (45)	*χ*^2^ = 2.24 *p* = 0.67	*χ*^2^ = 71.22 *p* = 0.004	*χ*^2^ = 8.42 *p* = 0.347	*χ*^2^ = 69.93 *p* = 0.001
Hospitalised owing to MLBP	09 (7)	*χ*^2^ = 4.54 *p* = 0.23	*χ*^2^ = 1.26 *p* = 0.11	*χ*^2^ = 1.46 *p* = 0.432	*χ*^2^ = 4.12 *p* = 0.49
Changed jobs or duties owing to MLBP	04 (3)	*χ*^2^ = 3.33 *p* = 0.41	*χ*^2^ = 5.45 *p* = 0.48	*χ*^2^ = 1.24 *p* = 0.752	*χ*^2^ = 2.23 *p* = 0.82
Reduced activity owing to MLBP	23 (19)	*χ*^2^ = 4.45 *p* = 0.52	*χ*^2^ = 6.12 *p* = 0.54	*χ*^2^ = 8.23 *p* = 0.744	*χ*^2^ = 33.22 *p* = 0.004
Seen a health professional owing to MLBP	28 (23)	*χ*^2^ = 9.33 *p* = 0.50	*χ*^2^ = 2.67 *p* = 0.43	*χ*^2^ = 5.44 *p* = 0.464	*χ*^2^ = 44.22 *p* = 0.034

MLBP, mechanical lower back pain.

Table 3 shows the prevalence of SIJD versus participants’ demographic profile. There were 62 (23%) participants who presented with SIJD during the physical screening. A moderate relationship was found between the SIJD and age (*χ*^2^ = 55.49, *p* = 0.003) and a poor relationship with golf experience (*χ*^2^ = 44.931, *p* = 0.005).

**TABLE 3:** Sacroiliac joint dysfunction versus participants’ demographic profile (*n* = 271).

Domain	Prevalence	Gender	Age	Race	Experience
SIJD	62 (23)	*χ*^2^ = 0.67 *p* = 0.67	*χ*^2^ = 55.49 *p* = 0.003	*χ*^2^ = 8.42 *p* = 0.344	*χ*^2^ = 44.93 *p* = 0.005

SIJD, sacroiliac joint dysfunction.

Sixty (23%) participants presented with both MLBP and SIJD and 146 (54%) presented with neither of the conditions (Table 4).

**TABLE 4:** Cross tabulation between mechanical lower back pain and sacroiliac joint dysfunction (*n* = 271).

Variables	MLBP		Total
	Yes (%)	No (%)	
**SIJD**			
Yes	60 (22)	2 (1)	62
No	63 (23)	146 (54)	209
**Total**	**123**	**148**	**271**

SIJD, sacroiliac joint dysfunction; MLPB, mechanical lower back pain.

A strong association between MLBP and SIJD was found (*χ*^2^ = 88.234, *p* = 0.0001), which indicated that golfers with MLBP also presented with SIJD.

## Discussion

There was a 22% prevalence of MLBP and SIJD and a strong association between them in these participants. The problem of MLBP negatively affected some golfers such that their physical activity level had to be reduced. Other studies on the prevalence of MLBP showed it to be 25%–34.5% among golfers, which is less than what this study showed (Finn 2013; Gosheger et al. 2003; McHardy, Pollard & Luo 2006; McHardy et al. 2007; Parziale & Mallon 2006; Reed & Wadsworth 2010; Wadsworth 2007). The prevalence of SIJD in this study (23%) fell within the range of SIJD prevalence reported among patients with lower back pain which is between 16% and 30% (Vanelderen et al. 2010). There is no literature that reports on the prevalence of SIJD among golfers. Our study also found that age and years of golf experience were moderately associated with MLBP and SIJD. Sutcliffe et al. (2008) reported that older golfers are more susceptible to MLBP and SIJD because of their poor spinal mobility and the ability to absorb forces applied to the spinal column.

Amateur golfers are believed to be at risk of lower back pain because they often train hard to become professional players (Evans et al. 2005). Major risk factors that contribute to their back pain are the poor endurance and strength of the trunk muscles (Evans et al. 2005). In a study conducted by Gosheger et al. (2003), out of 637 reported golf injuries, 92.3% were found to have back problems where excessive play was blamed. According to Hosea and Gatt (1996), the development of MLBP occurs because of poor swing mechanics and poor physical conditioning in amateur golfers. During the golf swing, the lumbar spine is under a variety of forces including compression, anterior-posterior shearing, torsion and lateral bending.

Most back pain develops gradually over time among golfers and occurs mostly during the follow through phase of the golf swing which contributes to increased pressure on the spine, especially in the modern golf swing (Finn 2013). Finn (2013) further states that the modern golf swing involves much separation of the hip and shoulder during a backswing and ends in hyperextension of the lumbar spine. There is an eccentric contraction of the abdominal muscles which slows trunk rotation and results in increased pressure on the annulus of the intervertebral disc during the modern golf swing phase (Finn 2013).

Studies indicate that SIJD is a significant source of lower back pain among golfers (Bulbulian. 2001; Finn 2013; Gluck et al. 2008; McHardy & Pollard 2005). Gluck et al. (2008) found that amateur golfers have multiple swing and bending inconsistences, leading to pain in the back as a result of poor swing mechanism. Problems can also be caused by weight-bearing forces associated with running or jumping (Tinmark et al. 2010). The SIJ is predominantly exposed to injury because it serves as a major link in the kinetic chain between the power generated above it and the stability provided below (Finn 2013; Tinmark, et al. 2010; Vanelderen et al. 2010). Pain that comes from the SIJ is mainly thought to be gluteal pain, which can then be referred to the lower and upper lumbar regions (Cohen et al. 2014; Vanelderen et al. 2010).

It is also important to note that the problems experienced by golfers are also costly to the golf industry, the workforce and the healthcare system (Reed & Wadsworth, 2010; Wadsworth 2007). Therefore, it is important for clinicians to conduct a thorough screening when managing these problems. A comprehensive rehabilitation approach is highly recommended when approaching golfers with these complex conditions. According to Finn (2013), a multidisciplinary team approach is the best strategy to prevent, rehabilitate and improve performance among golfers. Technical flaws and physical problems that lead to problems among golfers need to be addressed to prevent a continuous cycle of injuries (Finn 2013).

## Conclusion and recommendations

This study showed a 22% prevalence of MLBP and SIJD and a strong association between them. Therefore, injury prevention and effective rehabilitative programmes are critical in addressing these problems. Rehabilitation should include training of movement patterns, correcting muscle imbalances and type of swing because all these have a direct effect on the forces applied to the spine during the golf swing. Assessment and rehabilitation of SIJD is important when managing golfers with MLBP. A comprehensive multidisciplinary rehabilitation approach is highly recommended.

## References

[CIT0001] Batt M.E., 1992, ‘A survey of golf injuries in amateur golfers’, *British Journal of Sports Medicine* 26(1), 63–65. 10.1136/bjsm.26.1.63 1600459 PMC1478983

[CIT0002] Bulbulian R., 2001, ‘The short golf backswing: Effects on performance and spinal health implications’, *Manipulative Physical Therapy* 24(9), 569–575. 10.1067/mmt.2001.118982 11753330

[CIT0003] Cohen S.P., Chen Y. & Neufeld N.J., 2014, ‘Sacroiliac joint pain: A comprehensive review of epidemiology, diagnosis and treatment’, *Journal of Experts Review of Neurotherapeutics* 13(1), 99–116. 10.1586/ern.12.148 23253394

[CIT0004] Evans K., Refshaugea K., Adamsa R. & Aliprandic L., 2005, ‘Predictors of low back pain in young elite golfers: A preliminary study’, *Physical Therapy in Sport* 6(3), 122–130. 10.1016/j.ptsp.2005.05.003

[CIT0005] Finn C., 2013, ‘Rehabilitation of low back pain in golfers: From diagnosis to return to sport’, *Sports Health* 6(4), 313–319. 10.1177/1941738113479893 PMC3899905 24459546

[CIT0006] Gluck G.S., John A., Bendo J.M. & Spivak J.M., 2008, ‘The lumbar spine and low back pain in golf: A literature review of swing biomechanics and injury prevention’, *The Spine Journal* 8(5), 778–788. 10.1016/j.spinee.2007.07.388 17938007

[CIT0007] Gosheger G., Liem D., Ludwig K., Greshake O. & Winkelmann W., 2003, ‘Injuries and overuse syndromes in golf’, *American Journal of Sports Medicine* 31(3), 438–443. 10.1177/03635465030310031901 12750140

[CIT0008] Hayes A.F. & Krippendorff K., 2007, ‘Answering the call for a standard reliability measure for coding data’, *Communication Methods and Measures* 1(1), 77–89. 10.1080/19312450709336664

[CIT0009] Hosea T.M. & Gatt C.J. Jr, 1996, ‘Back pain in golf’, *Clinical Sports Medicine* 15(1), 37–53. 8903708

[CIT0010] Kirkaldy-Willis W.H. & Bernard T.N., 1999, ‘Making a specific diagnosis’, in *Managing low back pain*, 4th ed., pp. 206–226, Churchill Livingstone, Philadelphia, PA.

[CIT0011] Kuorinka I., Jousson B., Kilborn A., Vinterberg H., Biering-Sorensen F., Anderrsson G. et al., 1987, ‘Standardised Nordic questionnaires for the analysis of musculoskeletal symptoms’, *Applied Ergonomics* 18(3), 233–237. 10.1016/0003-6870(87)90010-X 15676628

[CIT0012] Laslett M., 2008, ‘Evidence-based diagnosis and treatment of the painful sacroiliac joint’, *The Journal of Manual & Manipulative Therapy* 16(3), 142–152. 10.1179/jmt.2008.16.3.142 19119403 PMC2582421

[CIT0013] Laslett M., Aprill C.N., McDonald B. & Young S.B., 2005, ‘Diagnosing painful sacroiliac joints: A validity of individual provocation tests and composites of tests’, *Manual Therapy* 10, 207–218. 10.1016/j.math.2005.01.003 16038856

[CIT0014] Laslett M., Young S.B., Aprill C.N. & McDonald B., 2003, ‘Diagnosing painful sacroiliac joints: A validity study of a McKenzie evaluation and sacroiliac provocation tests’, *Australian Journal of Physiotherapy* 49, 89–97. 10.1016/S0004-9514(14)60125-2 12775204

[CIT0015] Lindsay D.M. & Vandervoort A.A., 2014, ‘Golf-related low back pain: A review of causative factors and prevention strategies’, *Asian Journal of Sports Medicine* 5(4), e24289 10.5812/asjsm.24289 25741420 PMC4335481

[CIT0016] Maigne J.J., Aivaliklis A. & Pfefer F., 1996, ‘Results of sacroiliac joint double block & value of sacroiliac pain provocation tests in 54 patients with lower back pain’, *Spine Journal* 21(16), 1880–1892. 10.1097/00007632-199608150-00012 8875721

[CIT0017] McHardy A. & Pollard H., 2005, ‘Muscle activity during the golf swing’, *British Journal of Sports Medicine* 39(11), 799–804. 10.1136/bjsm.2005.020271 16244187 PMC1725059

[CIT0018] McHardy A., Pollard H. & Luo K., 2006, ‘Golf injuries: A review of the literature’, *Sports Medicine* 36(2), 171–187. 10.2165/00007256-200636020-00006 16464124

[CIT0019] McHardy A., Pollard H. & Luo K., 2007, ‘Golf-related lower back injuries: An epidemiological survey’, *Journal of Chiropractic Medicine* 6(1), 20–26. 10.1016/j.jcme.2007.02.010 19674690 PMC2647075

[CIT0020] Parziale J.R. & Mallon W.J., 2006, ‘Golf injuries and rehabilitation’, *Physical Medicine Rehabilitation Clinics of North America* 17(3), 589–607. 10.1016/j.pmr.2006.05.002 16952754

[CIT0021] Reed J.J. & Wadsworth L.T., 2010, ‘Lower back pain in golf: A review’, *Current Sports Medicine Reports* 9(1), 57–59. 10.1249/JSR.0b013e3181cab8ba 20071923

[CIT0022] Sutcliffe J., Ly J.Q., Kirby A. & Beall D.P., 2008, ‘Magnetic resonance imaging findings of golf-related injuries’, *Current Problem in Diagnostic Radiology* 37(5), 231–241. 10.1067/j.cpradiol.2007.08.005 18662601

[CIT0023] Tinmark F., Hellström J., Halvorsen K. & Thorstensson A., 2010, ‘Elite golfers’ kinematic sequence in full-swing and partial-swing shots’, *Sports Biomechanics* 9(4), 236–244. 10.1080/14763141.2010.535842 21309298

[CIT0024] Vanelderen P., Szadek K., Cohen S.P., De Witte J., Lataster A., Patijn J. et al., 2010, ‘Sacroiliac joint pain’, *Pain Practice* 10(5), 470–478. 10.1111/j.1533-2500.2010.00394.x 20667026

[CIT0025] Vleeming A., Schuenke M.D., Masi A.T., Carreiro1 J.E., Danneels L. & Willard F.H., 2012, ‘The sacroiliac joint: An overview of its anatomy, function and potential clinical implications’, *Journal of Anatomy* 221(6), 537–567. 10.1111/j.1469-7580.2012.01564.x 22994881 PMC3512279

[CIT0026] Waddell G., 1998, *The back pain revolution*, Churchill Livingstone, Edinburgh, UK.

[CIT0027] Wadsworth L.T., 2007, ‘When golf hurts: Musculoskeletal problems common to golfers’, *Current Sports Medicine Reports* 6(6), 362–365. 10.1097/01.CSMR.0000305613.90671.e8 18001607

[CIT0028] Weksler N., Velan G.J., Semionov M., Gurevitch B., Klein M., Rozentsveig V. et al., 2007, ‘The role of sacroiliac joint dysfunction in the genesis of low back pain: The obvious is not always right’, *Archives of Orthopaedic and Trauma Surgery* 127, 885–888. 10.1007/s00402-007-0420-x 17828413

